# Determinants of vitamin D status among Black and White low-income pregnant and non-pregnant reproductive-aged women from Southeast Louisiana

**DOI:** 10.1186/s12884-019-2246-2

**Published:** 2019-04-02

**Authors:** Natalie L. Burke, Emily W. Harville, Jeffrey K. Wickliffe, Arti Shankar, Maureen Y. Lichtveld, Michael L. McCaskill

**Affiliations:** 1 Department of Global Environmental Health Sciences, Tulane University School of Public Health and Tropical Medicine, 1440 Canal Street, Suite 2100, New Orleans, Louisiana 70112 USA; 2 Department of Epidemiology, Tulane University School of Public Health and Tropical Medicine, 1440 Canal Street, Suite 2001, New Orleans, LA 70112 USA; 3 Department of Global Biostatistics and Data Science, Tulane University School of Public Health and Tropical Medicine, 1440 Canal St, Room 2007, New Orleans, LA 70112 USA

**Keywords:** Vitamin D deficiency, Pregnancy, Women and infant clinics, Race, Sun exposure, Low income

## Abstract

**Background:**

Vitamin D deficiency is a growing public health problem, with pregnant women being particularly vulnerable due to its influences on maternal and neonatal outcomes. However, there are limited data published about mediators of vitamin D status in Louisiana women. We aimed to assess the vitamin D status and its determinants among low-income pregnant and non-pregnant reproductive-aged women from southeast Louisiana.

**Methods:**

This study was conducted using data from the Gulf Resilience on Women’s Health (GROWH) research consortium cohort of pregnant and non-pregnant women which contained sociodemographic and dietary variables as well as blood and salivary element concentrations. Serum 25-hydroxy vitamin D was measured using an enzyme-linked immunosorbent assay in 86 pregnant and 98 non-pregnant women with an even distribution of race in both groups.

**Results:**

The prevalence of deficient vitamin D levels in the total cohort (184 women) was 67% and the mean 25(OH) vitamin D_3_ was 24.1 ng/mL (SD 10.7). Self-identifying as White, being pregnant, autumn season, young age and high exposure to tobacco smoke measured by cotinine were significantly associated with higher serum levels of vitamin D. Visiting Women and Infant clinics (WIC) was an important determinant in improving 25(OH) vitamin D_3_ levels for Black women but not for White women and concentrations varied more among Black women across seasons compared to White women.

**Conclusions:**

Serum vitamin D levels are inadequate among a high proportion of Black and White low-income pregnant and reproductive-aged women living in Southeast Louisiana who were enrolled in the GROWH study. Black women who are over 35 years old and non-WIC participants constitute the subpopulation most at risk for vitamin D deficiency, especially during the winter. As an overall higher level of deficiency exists in Black women, if even small behavioral and dietary modifications are produced by WIC, this can lead to a comparatively greater improvement in vitamin D status in women from Southeast Louisiana who self-identify as Black.

## Background

Vitamin D is a nutrient and a prohormone that is gaining much attention in the medical literature due to the increasing understanding of its importance in health and disease. While vitamin D’s classical role is in calcium and bone homeostasis, the vitamin D receptor is present in most tissues, including the placenta, and it is involved in various biological actions, including cell proliferation and differentiation in many target tissues [1–3]. In adults, vitamin D deficiency has been linked to a number of adverse health outcomes, including increased risk of cardiovascular disease, infection, cancer, and even mortality [3–7]. During pregnancy, vitamin D readily diffuses across the placental barrier, and fetal and newborn vitamin D status is almost entirely dependent on vitamin D from the mother [8–10]. Pregnancy-related adverse health outcomes associated with vitamin D deficiency include gestational hypertension/preeclampsia [11–13], gestational diabetes [14], small for gestational age (SGA) [15], cesarean delivery [16] and, most recently, autism in the infants [17]. Furthermore, low vitamin D levels are associated with depressive symptoms during pregnancy and postpartum depression [16].

“Vitamin D” refers to two biologically inactive precursors: cholecalciferol (D_3_) and ergocalciferol (D_2_). The D_3_ form is obtained from synthesis in the skin under the influence of sunlight (ultraviolet B radiation (UVB); wavelengths 290–315) and dietary sources such as fatty fish and supplements. D_2_ is synthetically derived through the ultraviolet irradiation of ergosterol from yeast and fungi, such as mushrooms, and used to make vitamin D_2_ supplements. Both vitamin D_3_ and vitamin D_2_ are converted in the liver to circulating 25-hydroxyvitamin D_3_ [25(OH) D_3_], also known as calcidiol [1]. Therefore, 25(OH) D_3_ levels reflect the dietary input and/or cutaneous production of vitamin D_3_. This metabolite is not active but serves as a circulating reservoir that is further metabolized mostly in the kidneys into the active form, calcitriol [1,25(OH)D_3_]. Although 1, 25-Dihydroxyvitamin D_3_ is the biologically active form of vitamin D, 25(OH) D_3_ is the major form of circulating vitamin D and is highly stable for analysis from stored serum or plasma. This biological stability is due to its long half-life of about 2 to 3 weeks in serum, its high serum level, and non-tight regulation of 25 (OH) D_3_ formation [18–21]. Thus, most of the current literature uses 25(OH) D_3_ as the biomarker of overall vitamin D status.

Ultra-violet radiation mediated conversion of 7-dehydrocholesterol into vitamin D_3 _in the epidermal layer of the skin is the primary determinant of vitamin D status in most humans. The efficiency of the dermal conversion of 7-dehydrocholesterol is a function of type of skin pigmentation and the solar zenith angle which depends on latitude, season, and time of the day [22]. The skin pigment melanin limits the rate at which UVB radiation transmits through the skin, which reduces 7-dehydrocholesterol conversion. Consequently, individuals with darker skin pigment require more sun exposure to produce similar amounts of vitamin D_3_ as compared to those with less pigmented skin [22, 23]. It has been shown that persons with deeply pigmented skin and those living in high latitude locations during the winter season are particularly at risk of vitamin D deficiency [2, 22, 24].

Dietary sources of vitamin D generally contain modest amounts of the vitamin [3, 9]. Foods that naturally contain vitamin D include fatty fish, mushrooms, and egg yolk. Fortified foods include milk, orange juice, yogurts, butter, margarine, cheeses, and breakfast cereal [1, 8, 22]. Supplementation such as prenatal multi-vitamins containing vitamin D_3_ or D_2_, or of vitamins containing D_3_ or D_2_ alone can directly influence systemic vitamin D levels. While the administration of prenatal vitamins is primarily to maintain healthy levels of folic acid, iron, and calcium, most of prenatal vitamins also contain around 400 IU–600 IU of vitamin D_3_ [25]. Prenatal supplementation may partially explain why  pregnant women tend to have higher 25(OH) D_3_ levels compared to non-pregnant women [24]. Additional “influencing factors” that have been associated with vitamin D deficiency include obesity, high education and low age [2, 26].

While vitamin D deficiency is an important public health problem [26, 27], to our knowledge there is only one published research article  describing vitamin D health in pre or post partum women living in coastal communities in states bordering the Gulf of Mexico [28]. Data shows that reproductive-aged women are vulnerable to vitamin D deficiency, which is associated with many adverse reproductive outcomes [3, 26–28]. Dietary intake and/or supplementation is crucial to maintaining a healthy vitamin D status when vitamin D_3_ synthesized from sunlight is limited. Hence, the goal of this project was to provide a greater understanding of circulating vitamin D_3_ levels and to determine related risk factors such as heavy metal exposure, cigarette smoke exposure, age and race, in a population at high risk of vitamin D deficiency [29].

## Methods

### Study design and population

This study is embedded in the Transdisciplinary Research Consortium for Gulf Resilience on Women’s Health (GROWH). GROWH is a National Institute of Health-funded research consortium designed to address health issues of concern to the residents of the Gulf of Louisiana parishes affected by the Deepwater Horizon (DWH) oil spill, focusing on women of reproductive age [29–31]. The long-term goal of GROWH is to examine the causes of adverse reproductive outcomes and ultimately develop interventions to improve those outcomes. GROWH supported population-based and laboratory research to develop the scientific evidence base needed to promote health and well-being for people living along the Gulf Coast who are at greatest risk for potential adverse physical, psychological and behavioral health effects.

Between January 2011 and December 2016, approximately 1700 women who fulfilled the inclusion criteria of being 18–45 years old or pregnant and older than 45, living in one of the affected parishes at the time of the oil spill (2010), singleton pregnancy, and English-, Spanish-, or Vietnamese-speaking were recruited from prenatal Women, Infants, and Children (WIC) clinics, primary care clinics as well as community organizations in Southern Louisiana. The Special Supplemental Nutrition Program for WIC serves low-income pregnant and postpartum women and provides nutrition education and food vouchers. WIC programs do not provide direct prenatal care services but do reach a large proportion of low-income women during the perinatal period [32].

The recruitment of this population was done through convenience sampling at a wide range of health and community facilities, which likely makes it more representative of exposure and effects in the community compared to studies of litigants or those referred for clinical evaluation [30, 33]. Although details of prenatal vitamin use were not collected, all women received prenatal care and standard prenatal care calls for prescription of prenatal vitamins (data not shown), which typically contain 400 IU–600 IU vitamin D [22, 25]. In previous studies of similar populations > 90% of women reported regularly taking prenatal vitamins [34].

### Sample

**Fig. 1:**
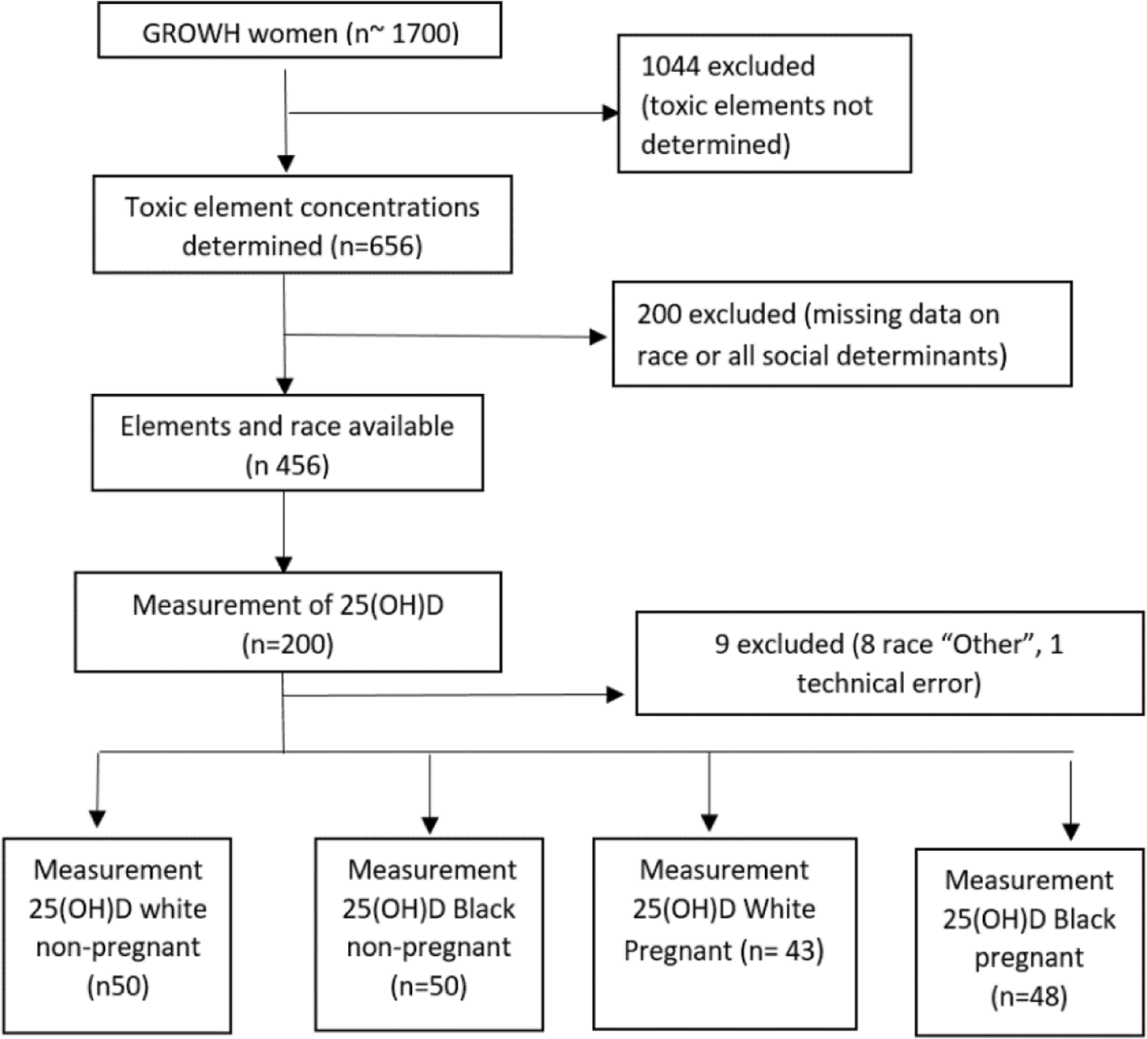
A flow chart illustrating the inclusion and exclusion criteria of the study population selection

The current study involved a sub-cohort of the overall 1700 GROWH cohort. The sample consisted of 91 pregnant women (48 Black and 43 White) and 100 non-pregnant women of reproductive age (50 Black and 50 White) who were randomly selected based on two inclusion criteria (Fig.1). The sample was limited to those with measured elemental concentrations (Lead [Pb], Mercury [Hg], Cadmium [Cd]) and reported race data. The race category “Black” consists of non-Hispanic Black as well as Hispanic, Black women. The race category “White” consists of non-Hispanic White as well as Hispanic, White women.

Circulating blood serum 25(OH) vitamin D concentrations were determined for 100 pregnant women and 100 non-pregnant women. Participants were randomly selected within racial groups to provide an even distribution in the pregnant and non-pregnant groups. Nine measurements were excluded from the study due to incorrect race categorization and one case of vitamin D concentration below the level of detection due to a lab error.

### Biomarkers

Maternal venous blood was drawn at the same visit as the interview and questionnaire, at the convenience of the participants. All blood samples were processed within 12 h, and aliquots of serum or plasma were frozen at − 70° Celsius until analysis.

Biomarker analyses of elements Pb, Cd, Hg from serum and salivary cotinine (a biomarker for tobacco use) were performed for a random sample of pregnant and non-pregnant women of the GROWH cohort. Metals were analyzed using inductively coupled plasma-mass spectrometry (ICP-MS). Standards and blank tubes underwent the same digestion procedures as the samples. The concentration of each metal analyzed in whole blood samples is expressed as micrograms per liter or micrograms per deciliter where appropriate [35].

Elements were investigated as continuous as well as dichotomous variables. None of the women in this sample had levels exceeding CDC or OSHA guidelines for lead or cadmium (5 μg/dL and five μg/L), so we choose to dichotomize based on median values. For mercury, we adhered to the recommendations of the US Environmental Protection Agency (EPA) of 5.8 μg/L for cord blood [36, 37].

Cotinine was investigated as a continuous variable and as a dichotomous variable to distinguish women with low tobacco smoke exposure from women with high levels of tobacco smoke exposure, using the 12.9 ng/mL cutoff for pregnant women determined by Stragierowics et al., 2013 [38]. Cotinine represents total tobacco exposure, meaning environmental tobacco smoke exposure as well as from first-hand tobacco smoking.

### Assessment of circulating 25(OH) vitamin D_3_ concentration

For this study, the GROWH serum samples collected between 2011 and 2016 were thawed, and 25-Hydroxyvitamin D [25(OH)D_3_], which is highly stable for analysis was measured in serum. Although serum vitamin D levels have been correlated with many diverse health endpoints, Vieth et al. 2006 emphasizes that often the criteria for vitamin D adequacy are promulgated from randomized clinical trials investigating associations between vitamin D and bone health. Garland et al. 2014 concluded that serum 25(OH) vitamin D_3_ concentrations greater than 30 ng/mL resulted in lower all-cause mortality than concentrations less than or equal to 30 ng/mL (*p* < .01) [3]. Acknowledging the growing body of evidence that vitamin D_3_ levels > 30 ng/mL (75 nmol/L) are required for optimum overall health [3, 7, 39], we adhered to this as a cutoff measurement.

This measurement was performed using the Total 25-OH vitamin D_3_ ELISA analysis kit from Crystal Chem (Downers Grove, Illinois, U.S.A.), following the manufacturer’s recommended concentrations and procedures. Subsequently, 25(OH) vitamin D_3_ concentrations were determined using GraphPad Prizm (7.0) software. The total 25(OH) vitamin D_3_ calibration curve was constructed by plotting the mean absorbance values for each standard on the Y-axis against the corresponding 25(OH) vitamin D_3_ concentration on the X-axis. A four-parameter logistic (4-PL) curve was used for evaluation.

### Determinants of vitamin D status

**Fig. 2:**
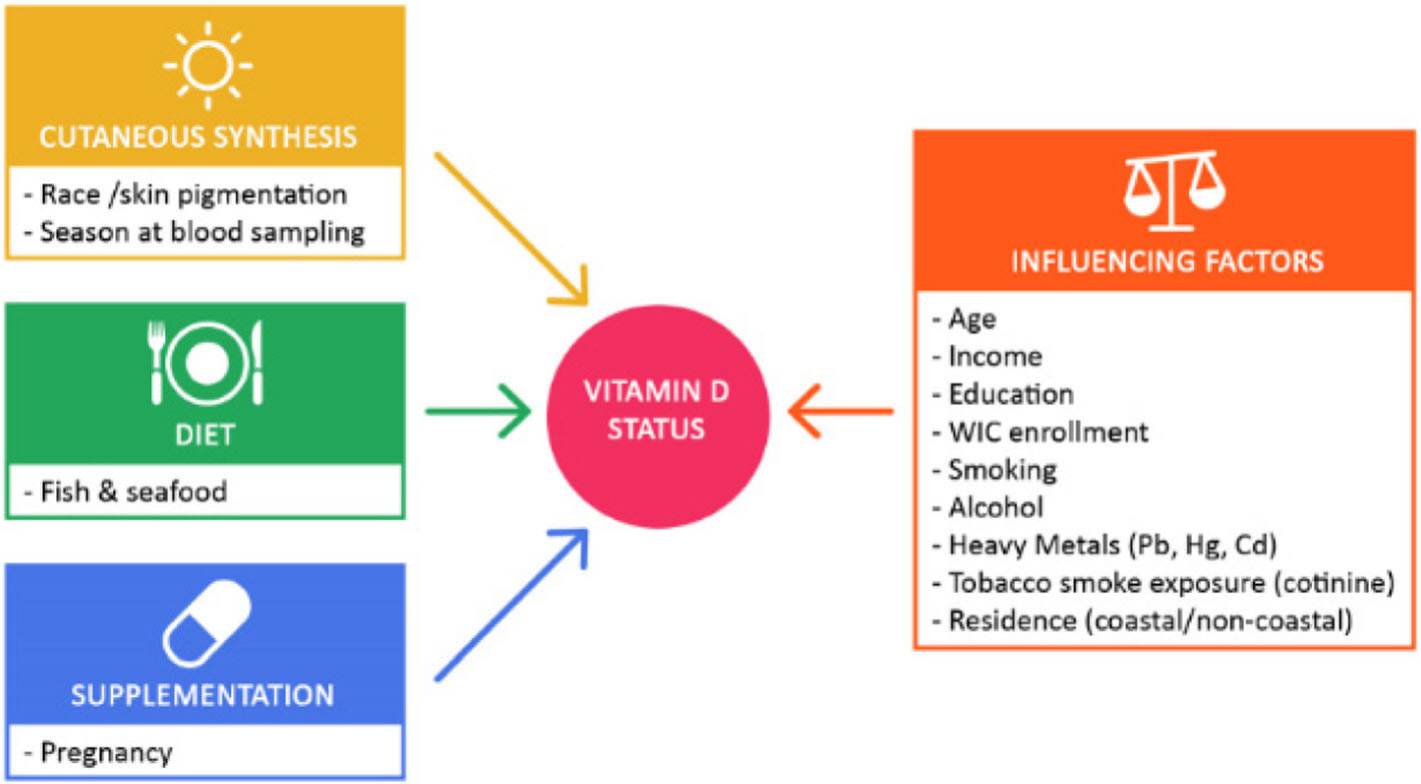
Determinants investigated for associations with serum vitamin D_3_ levels

We investigated dietary, socioeconomic, and environmental factors for associations with vitamin D (Fig. 2**);** race, season at blood sampling, pregnancy, age, income class, education level, enrollment in WIC clinics, pre-pregnancy BMI, smoking levels and alcohol consumption, and fish and seafood intake.

Seasons were calculated based on interview dates and were defined as follows: spring (March through May), summer (June through August), autumn (September through November), winter (December through February). WIC status was determined based on the name of the clinic of enrollment.

### Measurement instruments

#### Demographics

A short series of questions examining the demographics and social status of the study participants was created for the GROWH study. Questions chosen from the scale included those investigating race, age, BMI, income levels, education levels, and residence.

#### Smoking and alcohol use

Questions regarding health behavior and access to prenatal care for the GROWH study were adapted from PRAMS, the Pregnancy Risk Assessment Monitoring [40]. Smoking status was based on the question: “In the *three months before* you got pregnant, how many cigarettes did you smoke on an average day? ”Alcohol use was based on the question: “During the *three months before* you got pregnant, how many alcoholic drinks did you have in an average week?”

#### Fish consumption

The Fish Food Frequency Questionnaire by Mina et al. 2007, was adapted for local fish [41, 42]. This study focused on fish and seafood known to contain relatively high vitamin D levels [1, 8, 22], and those most frequently consumed by the study population. Fish considered to be high in vitamin D were: canned tuna, canned salmon, tuna (not canned), salmon (not canned), White trout, cod, Spanish mackerel, king mackerel, mahi-mahi, and swordfish. These fish were all grouped into the category fatty fish meaning that if a participant ate any of the before mentioned fish, it would add to the frequency of fatty fish consumption. The frequency was multiplied by the ounces of fish the women regularly ate per serving. Fatty fish consumption was analyzed as a categorical variable (0 oz per week, 0–3.5 oz per week, more than 3.5 oz per week) and as a continuous variable. The most frequently consumed fish and seafood were analyzed as a continuous variable, looking at mean servings per week.

#### Pre-pregnancy body mass index (BMI)

Body mass index was based on self-reported height at recruitment and pre-pregnancy self-reported weight (kg/m2) (underweight (< 18.5), normal weight (18.5–24.99), overweight (25–29.99), and obese (> 30).

### Statistical analysis

The Statistical Package for Social Sciences (SPSS Inc. version 22) was used for data analysis. First, all data were analyzed descriptively via univariate analysis. Vitamin D status was expressed as a categorical variable to assess the prevalence and degree of deficiency among the entire study population and as a continuous variable (means + standard deviations) for bivariate analysis and linear regression. The distributions for serum vitamin D concentrations, heavy metals levels, and fish and seafood consumption were explored by assessing normality plots and by conducting tests of normality including the Kolmogorov-Smirnov test and the Shapiro-Wilk test. After serum vitamin D concentrations showed a non-normal distribution, outliers were identified by adding or subtracting twice the standard deviation (2 × 12.9) to the mean vitamin D levels (25.6 ng/mL). Seven outliers with 25(OH) D_3_ levels ranging from 53 ng/mL to 75 ng/mL were removed after which the distribution of vitamin D levels became normal according to normality plots and tests of normality. Six of the seven outliers where White. Five where pregnant, and four had a coastal zip code. Heavy metals and fish and seafood consumption were non-normally distributed and were analyzed using non-parametric tests.

Second, bivariate associations between the dependent variable 25(OH) D_3_ and categorical variables such as race, income, age were examined using independent sample t-test and one-way ANOVA (with Tukey’s post hoc test). Kruskal- Wallis Test or Mann-Whitney U test was used for nonparametric analyses. Associations of vitamin D with continuous variables was done using Pearson correlation and Spearman correlation if the continuous variable was not normally distributed. Due to the established relationship between race and vitamin D status, we chose to investigate interactions between race and the other covariates. To investigate interactions between two independent variables on vitamin D levels, a factorial design (two-way ANOVA) was used.

Predictor variables associated with the dependent variable at a statistically significant level (*p* < 0.05) were entered in the final multiple regression model multivariate model. Third, multiple linear regression was used to estimate the association between 25(OH)D_3_ and the included predictor variables. The standardized beta coefficient and 95% confidence intervals were reported.

## Results

Of the GROWH 2011–2016 participant cohort, we analyzed the characteristics of a sample of 184 women (84 pregnant, 100 non-pregnant). Characteristics of the study population are presented in Table 1. Fish and seafood most frequently consumed among this population were shrimp followed by canned tuna, crab, catfish, crayfish and tilapia (data not shown).

**Table 1:** Characteristics of all women, pregnant women vs. non-pregnant women and Black vs White women

Characteristics	All women *n* = 184 ^*^(%)	Pregnant women *n* = 84^*^ (%)	Non-pregnant women *n* = 100^*^(%)	P)	White women *n* = 87^*^ (%)	Black women *n* = 97^*^(%)	P^a^)
Race				0.623			
Black	97 (53%)	47 (55%)	50 (51%)		–	–	
White	87 (47%)	39 (45%)	48 (49%)		–	–	
Season				0.263			0.199
Spring	31 (18%)	14 (17%)	17 (19%)		14(17%)	17(19%)	
Summer	84 (49%)	35 (43%)	49 (54%)		34(42%)	50(55%)	
Autumn	43 (25%)	24 (29%)	19 (21%)		26(32%)	17(19%)	
Winter	14 (8%)	9(11%)	5 (6%)		7(9%)	7(8%)	
Fish consumption				0.663			0.206
0 oz/week	50 (27%)	25 (29%)	25 (26%)		29 (33%)	21 (22%)	
0–3.5 oz/week	90 (49%)	39 (45%)	51 (52%)		39 (45%)	51 (53%)	
> 3.5 oz/week	44 (24%)	22 (26%)	22 (22%)		19 (22%)	25,926%)	
Pregnant							0.623
no	98 (53%)	–	–		48 (55%)	50 (52%)	
yes	86 (47%)	–	–		39 (45%)	47 (48%)	
Age				**0.020**			0.952
18–25	68 (40%)	38 (47%)	30 (33%)		31 (39%)	37 (40%)	
> 25–30	46 (27%)	25 (31%)	21 (23%)		21 (26%)	25 (27%)	
> 30–35	35 (20%)	12 (15%)	23 (25%)		16 (20%)	19 (21%)	
> 35	23 (13%)	6 (7.4%)	17 (19%)		12 (15%)	11 (12%)	
BMI				0.243*			0.618*
< 18.5	2 (1%)	2 (2%)	0 (0%)*		0 (0%)*	2(2%)	
18.5-24.99	47 (26%)	24 (30%)	23(24%)		22 (27%)	25(26%)	
25–29.99	43 (24%)	16 (20%)	27(28%)		20(24%)	23(24%)	
> 30	86 (48%)	39 (48%)	47 (49%)		41 (49%)	45(47%)	
Income				0.899			0.417
< $ 10,000	85 (50%)	38 (48%)	47 (52%)		35 (46%)	50 (54%)	
>$ 10,000 - $ 30,000	56 (33%)	27 (34%)	29 (32%)		26(34%)	30 (32%)	
> $ 30,000	29 (17%)	14 (18%)	15 (17%)		16 (21%)	13 (14%)	
WIC				**0.003**			0.684
yes	121 (69%)	50 (58%)	71 (79%)		59 (70%)	62 (67%)	
no	55 (31%)	36 (42%)	19 (21%)		25 (30%)	30 (33%)	
Education level				0.793			0.641
High school or less	96 (53%)	45 (54%)	51 (52%)		47(55%)	49 (51%)	
Some college/Associate’s degree	77 (42%)	34 (40%)	43 (44%)		35(41%)	42 (43%)	
College graduate or higher	9 (5%)	5 (6%)	4 (4%)		3(4%)	(6%)	
Smoking level				**0.000**			**0.000**
nonsmoker	114 (64%)	54 (66%)	60 (63%)		41 (50%)	73 (77%)	
former	16 (9%)	14 (17%)	2 (2%)		11(13%)	5 (5%)	
1–10 cigs/day	39 (22%)	14 (17%)	25 (26%)		23 (28%)	16 (17%)	
> = 11 cigs/day	8 (5%)	0 (0%)*	8 (8%)		7 (9%)	1 (1%)	
Cotinine				0.547			**0.013**
High (> 12.9 ng/mL)	21 (29%)	12 (27%)	9 (33%)		15(43%)	6 (16%)	
Low (< 12.9 ng/mL)	51 (71%)	33 (73%)	18 (67%)		20 (57%)	31 (84%)	
Lead (Median)				0.052			0.596
High (> 0.48μg/dL)	85 (50%)	30 (41%)	55 (56%)		39 (52%)	47 (48%)	
Low (< 0.48μg/dL)	86 (50%)	43 (59%)	43 (44%)		36 (48%)	50 (52%)	
Mercury				0.215			0.126
High (> 5.8 μg/L)	89 (52%)	42 (58%)	47 (48%)		44 (59%)	45 (47%)	
Low (< 5.8 μg/L)	82 (48%)	31 (43%)	51 (52%)		31 (41%)	51 (53%)	
Cadmium (median)				0.058			0.381
High (>.47 μg/L)	87 (51%)	31 (42%)	56 (57%)		41 (55%)	46 (48%)	
Low (<.47 μg/L)	84 (49%	42 (58%)	42 (43%)		34 (45%)	50 (52%)	
Proximity to coast				**0.012**			**0.014**
Zip code proximal to coast	30 (18%)	8 (10%)	22 (25%)		21 (25%)	9(11%)	
Further from coast	139 (82%)	72 (90%)	67 (75%)		63(75%)	76(89%)	

^*^Data may not add to total amounts due to missing data

p) The *p*-values refer to differences between pregnant and non-pregnant women, using Pearson chi-square tests (Bold type indicates significance *p* < 0.05

p^a^) The *p*-values refer to differences between White and Black women, using Pearson chi-square tests (Bold type indicates significance  *p* < 0.05)

*Chi-square test not reliable if cell has frequency of 0

### Population characteristics by pregnancy status

The characteristics that differed significantly between pregnant and non-pregnant women included age, WIC participation, self-reported smoking levels, and proximity to the coast. Pregnant women who were younger were not recruited through WIC clinics as often as non-pregnant women. Younger study participants reported lower rates of smoking cigarettes and most did not live close to the coast. Although pregnant women reported less smoking behavior, cotinine concentrations were similar between the pregnant and non-pregnant group.

### Population characteristics by race

Between Black and White women, there was a difference in self-reported smoking levels with White women smoking more than Black women. Cotinine levels confirm this result.

Vitamin D status and mean serum 25(OH) vitamin D_3_ levels are presented in Table 2. Overall, 38% of the women had serum 25(OH) D_3_ concentrations < 20 ng/mL (severe deficiency) and 29% had concentrations between 21 and 30 ng/mL (moderate deficiency). Levels below 20 ng/mL were much more prevalent in Black women than White women (52% vs. 23%). In pregnant women, 26% had levels < 20 ng/mL compared to 49% in the non-pregnant group. Women sampled in the autumn had lower levels of vitamin D deficiency than other seasons (16%).

**Table 2:** Vitamin D status (severe deficiency/moderate deficiency) and mean 25(OH)D levels stratified by characteristics including significance values for bivariate analyses

Characteristics	Severe deficiency (< 20 ng/mL)	Moderate deficiency (21–30 ng/mL)	p	Mean 25(OH)D levels (SD)	P^a^
Total	38%	29%		24.1 (10.7)	
Race			**0.000**		**0.000**
Black	52%	29%		20.7 (9.9)	
White	23%	29%		27.9 (10.4)	
Season			**0.048**		**0.019**
Spring	42%	32%		22.0 (10.8)*	
Summer	44%	26%		23.1 (10.8)*	
Autumn	16%	33%		28.8 (9.7)*	
Winter	43%	36%		23.0 (11.7)	
Fatty fish consumption			0.272		0.605
0 oz/week	26%	32%		26.8 (10.3)	
0–3.5 oz/week	44%	26%		22.9 (11.0)	
> 3.5 oz/week	39%	32%		23.5 (10.3)	
Pregnant			**0.005**		**0.016**
Yes *White* *Black*	26 *10%* *38%*	35 36% 33%		25.9 (11.3) 30.5(10.6)^b^ 22.1 (10.6)^b^	
No *White* *Black*	49 *33%* *64%*	24 22% 25%		22.6(10.0) 25.8(9.9)^b^ 19.5(9.2)^b^	
Age			0.056		**0.014**
18–25	27%	29%		27.0 (11.6) *	
> 25–30	37%	26%		24.1 (10.5)	
> 30–35	49%	34%		20.8 (8.6) *	
> 35	52%	30%		20.8 (9.5)	
BMI			**–**		0.754
< 18.5	–	–		29.1 (10.0)	
18.5–24.99	–	–		24.8 (11.7)	
25.29.99	–	–		22.8 (10.5)	
> 30	–	–		23.9 (10.5)	
Income			0.579		0.894
< $ 10,000	38%	33%		23.7 (10.1)	
>$ 10,000 - $ 30,000	45%	29%		23.3 (11.7)	
> $ 30,000	38%	21%		24.4 (11.1)	
WIC			0.282		0.120
yes	35%	31%		24.8 (10.4)	
no	47%	26%		22.8 (11.3)	
Education level			0.787		0.946
High school or less	37%	29%		24.2 (10.7)	
Some college/Associate’s degree	42%	26%		23.9 (11.0)	
College graduate or higher	33%	44%		25.0 (10.1)	
Alcohol use (drinks/week)-					0.146
14 or more	–	–		22.5 (12.7)	
7 to 13	–	–		26.2(6.8)	
4 to 6	–	–		22.6 (8.2)	
1 to 3	–	–		20.2 (10.9)	
Less than 1	–	–		27.5 (11.0)	
I didn’t drink then	–	–		23.9 (10.9)	
Smoking level			0.795		0.445
nonsmoker	42%	28%		23.2 (10.7)	
former	31%	38%		24.8 (11.3)	
1–10 cigs/day	31%	28%		26.3 (10.7)	
> = 11 cigs/day	38%	38%		23.0 (10.9)	
Cotinine			**0.013**		**0.005**
High (> 12.9 ng/mL)	16%	24%		29.9 (10.7)	
Low (< 12.9 ng/mL)	42%	31%		21.8 (11.0)	
Cadmium (median)			0.281		0.482
High (> 0.47 μg/L)	40%	33%		23.1 (9.8)	
Low (< 0.47μg/L)	41%	24%		24.2 (11.3)	
Lead (median)			0.257		0.211
High (> 0.48 μg/dL)	46%	28%		22.6 (10.5)	
Low (< 0.48 μg/dL)	35%	29%		24.7 (10.5)	
Mercury			0.168		0.446
High (> 5.8 μg/L)	24%	47%		25.5 (10.1)	
Low (< 5.8 μg/L)	42%	27%		23.5 (10.6)	
Proximity to coast			0.488		0.875
Zip code proximal to coast	43%	20%		23.8 (9.9)	
Further from coast	37%	31%		24.1 (11.1)	

*P* values determined by chi-square tests. Differences were considered statistically significant at *p* < 0.05, bold type indicates significance

p^a^ values determined by Independent-Samples T test or one-way ANOVA; significance at *p* < 0.05; bold type indicates significance

^*^significantly different using Tukey’s test

^b^significantly different using Independent-Samples T test

- due to small n in cells, chi-square test assumptions were not met and could not be performed

Interestingly, vitamin D deficiency was seen less in the high cotinine group. Other factors such as income level, WIC participation, education level, self-reported smoking levels, exposure to heavy metals, fatty fish consumption, and proximity to coast did not show any relationship with vitamin D status. The overall mean serum 25(OH) D_3_ level was 24.1 ng/mL. Vitamin D levels varied strongly between Black and White women with Black women having lower concentrations than White women (20.7 vs. 27.6 ng/mL) Serum 25(OH) D_3_ levels were significantly higher among pregnant women compared to non-pregnant women (25.9 vs. 22.6; *P* = .037). Other determinants that were associated with higher levels of vitamin D included being sampled in the autumn, having a young age, and belonging to the high cotinine group. Correlation tables revealed that vitamin D was not correlated with any of the heavy metals but was positively associated with cotinine levels as a continuous variable (Table 3). Furthermore, vitamin D was not associated with any of the most frequently consumed fish (Table 4**).**

**Table 3:** Spearman’s rho bivariate analysis of vitamin D with heavy metals

	Vit D (ng/mL)	Pb (μg/dL)	Hg (μg/L)	Cd (μg/L)	Salivary cotinine (ng/mL)
Vit D (ng/mL)					
Correlation Coefficient	1.000				
Sig. (2-tailed)	.				
Pb (μg/dL)					
Correlation Coefficient	− 0.099	1.000			
Sig. (2-tailed)	0.196	.			
Hg (μg/L)					
Correlation Coefficient	−0.013	0.099	1.000		
Sig. (2-tailed)	0.865	0.199	.		
Cd (μg/L)					
Correlation Coefficient	−0.033	0.364^b^	0.206^a^	1.000	
Sig. (2-tailed)	0.665	0.000	0.007	.	

^a^ Correlation is significant at the 0.05 level (2-tailed)

^b^ Correlation is significant at the 0.01 level (2-tailed)

**Table 4:** Spearman’s rho bivariate analysis of vitamin D with fish and seafood

	1.	2.	3.	4.	5.	6.	7.	8.
1. Vit D (ng/mL)								
Correlation Coefficient	1.000							
Sig. (2-tailed)	.							
2.Fatty fish consumption								
in ounces Correlation	−0.118	1.000						
Coefficient	0.111	.						
Sig. (2-tailed)								
3. Shrimp								
Correlation Coefficient	−0.119	0.212^b^	1.000					
Sig. (2-tailed)	0.125	0.006	.					
4. Canned Tuna								
Correlation Coefficient	−0.113	0.753^b^	0.284^b^	1.000				
Sig. (2-tailed)	0.143	0.000	0.000	.				
5. Crab								
Correlation Coefficient	−0.022	0.244^b^	0.713^a^	0.280^b^	1.000			
Sig. (2-tailed)	0.782	0.002	0.000	0.000	.			
6. Catfish								
Correlation Coefficient	−0.103	0.348^b^	0.304^b^	0.360^b^	0.262^a^	1.000		
Sig. (2-tailed)	0.187	0.000	0.000	0.000	0.001	.		
7. Crayfish								
Correlation Coefficient	−0.036	0.386^b^	0.370^a^	0.325^b^	0.447^a^	0.199^a^	1.000	
Sig. (2-tailed)	0.648	0.000	0.000	0.000	0.000	0.012	.	
8. Tilapia								
Correlation Coefficient	−0.096	0.517^b^	0.234^b^	0.403^b^	0.214^b^	0.377^b^	0.262^b^	1.000
Sig. (2-tailed)	0.220	0.000	0.002	0.000	0.007	0.000	0.001	.

^a^ Correlation is significant at the 0.05 level (2-tailed)

^b^ Correlation is significant at the 0.01 level (2-tailed)

**Fig. 3:**
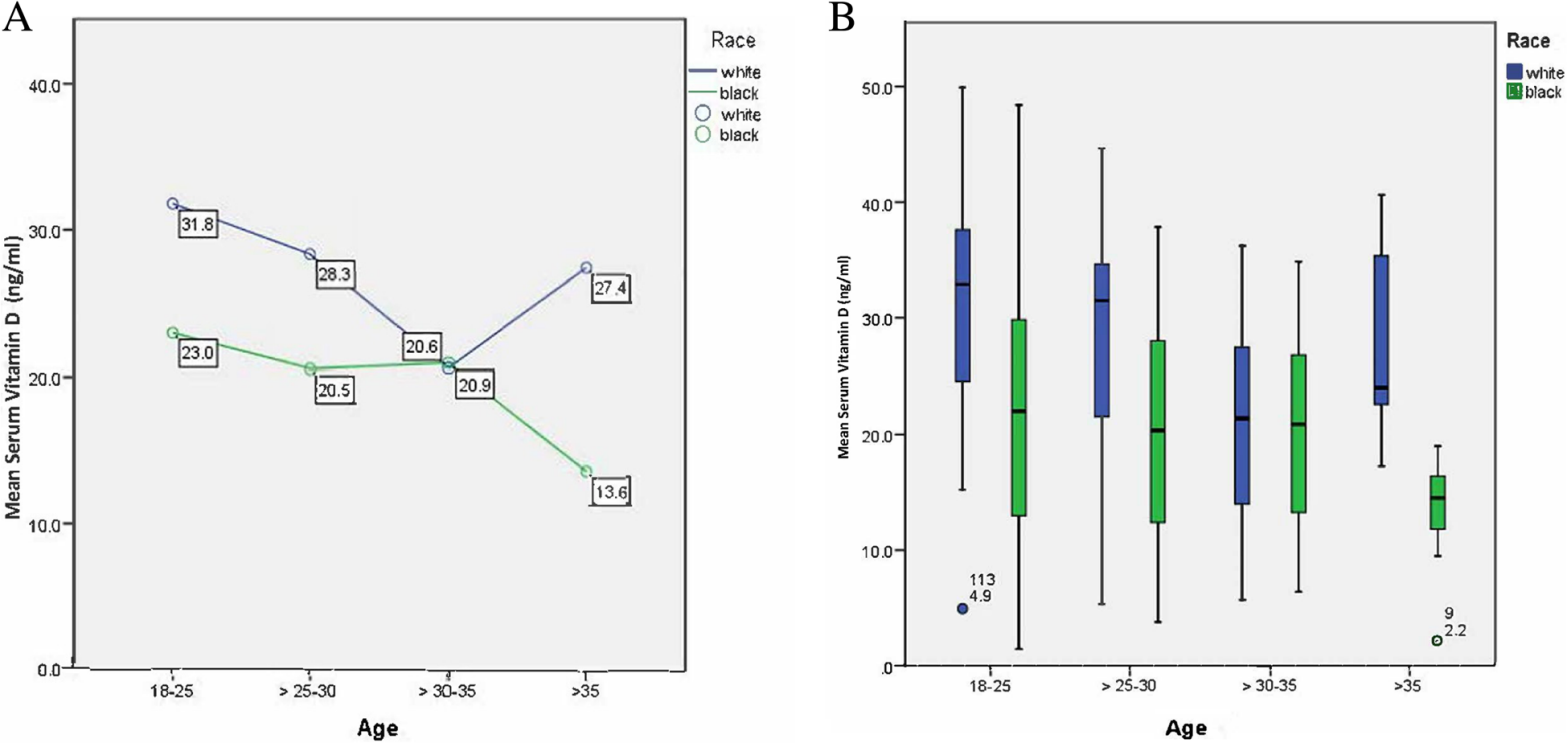
**a** and **b** The influence of race and age on mean serum vitamin D_3_ levels in surveyed women

**Fig. 4:**
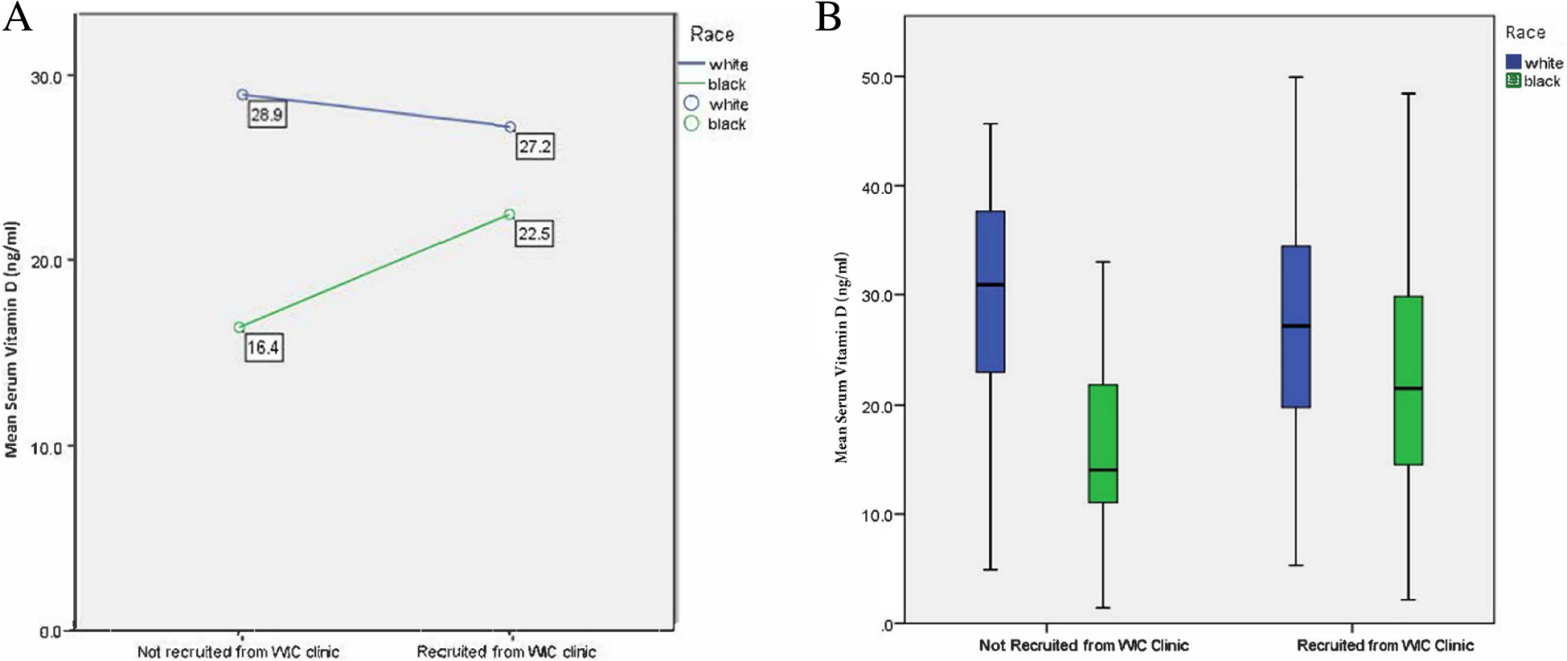
**a** and **b** The influence of race and location of recruitment site on mean serum vitamin D_3_ levels in surveyed women

 Multivariable predictors of 25(OH)D levels (continuous) are presented in Table 5. Race was the strongest predictor for mean 25(OH)D levels followed by season, age, and pregnancy respectively. The covariates that interacted with race in determining mean 25(OH) D_3_ levels included age and WIC clinic use (Figs. 3 and 4). For all the age categories, except 30–35, White women had a significantly higher mean serum 25(OH) D_3_ than Black women (Fig. 3 ). The sudden dip in vitamin D in White women in age category 30–35 brings mean serum 25(OH) D_3_ levels very close to that of Black women (20.6 vs. 20.9 respectively). Women in the youngest age group had the highest vitamin D levels in White as well as Black women (31.8 ng/mL vs. 23.0 ng/mL). For White women, those in age category 30–35 had the lowest vitamin D levels (20.6 ng/mL) while it was age category > 35 for Black women (13.6 ng/mL). The largest difference in mean serum 25(OH) D_3_ levels between Black and White women is observed in age category > 35 (13.6 ng/mL Black vs. 27.4 ng/mL White; *p* < 0.001). Post-hoc analysis showed that all Black women in the age category > 35 years were non-pregnant. In the White group aged over 35, there were three pregnant women versus 13 non-pregnant.

**Table 5:** Linear regression model for analysis of mean 25(OH)D levels with race, pregnancy, season, age, WIC status in the study population

Characteristics	β (CI 95%)
Race	
Black	−6.8 (− 9.8 to − 3.7)
White	Referent
Pregnant	
Yes	3.4 (0.2 to 6.6)
No	Referent
Season	
Spring	−5.7 (−10.4 to − 1.0)
Summer	−3.4 (−7.1 to 0.4)
Autumn	Referent
Winter	−6.1 (−12.5 to 0.4)
Age	
18–25	Referent
> 25–30	−3.0 (−6.8 to 0.8)
> 30–35	−5.0 (−9.2 to - 0.9)
> 35	−4.3 (−9.2 to 0.7)
WIC	
Yes	2.4 (−1.0 to 5.8)
No	Referent

CI, confidence interval

While being enrolled in the WIC program did not independently influence serum 25(OH) D_3_ levels, it does influence the effect of race on vitamin D status (*p* = .017) (Fig. 4). White women selected from WIC clinics did not have significantly different mean serum vitamin D levels than White women not from WIC clinics (27.2 ng/mL vs. 28.9 ng/mL respectively). However, Black women from WIC clinics had significantly higher mean vitamin D levels than those not from WIC clinics (22.5 ng/mL vs. 16.3 ng/mL). This observation was not due to the influence of pregnancy (Black pregnant and non-pregnant women were enrolled at WIC clinics in similar proportions).

**Fig. 5:**
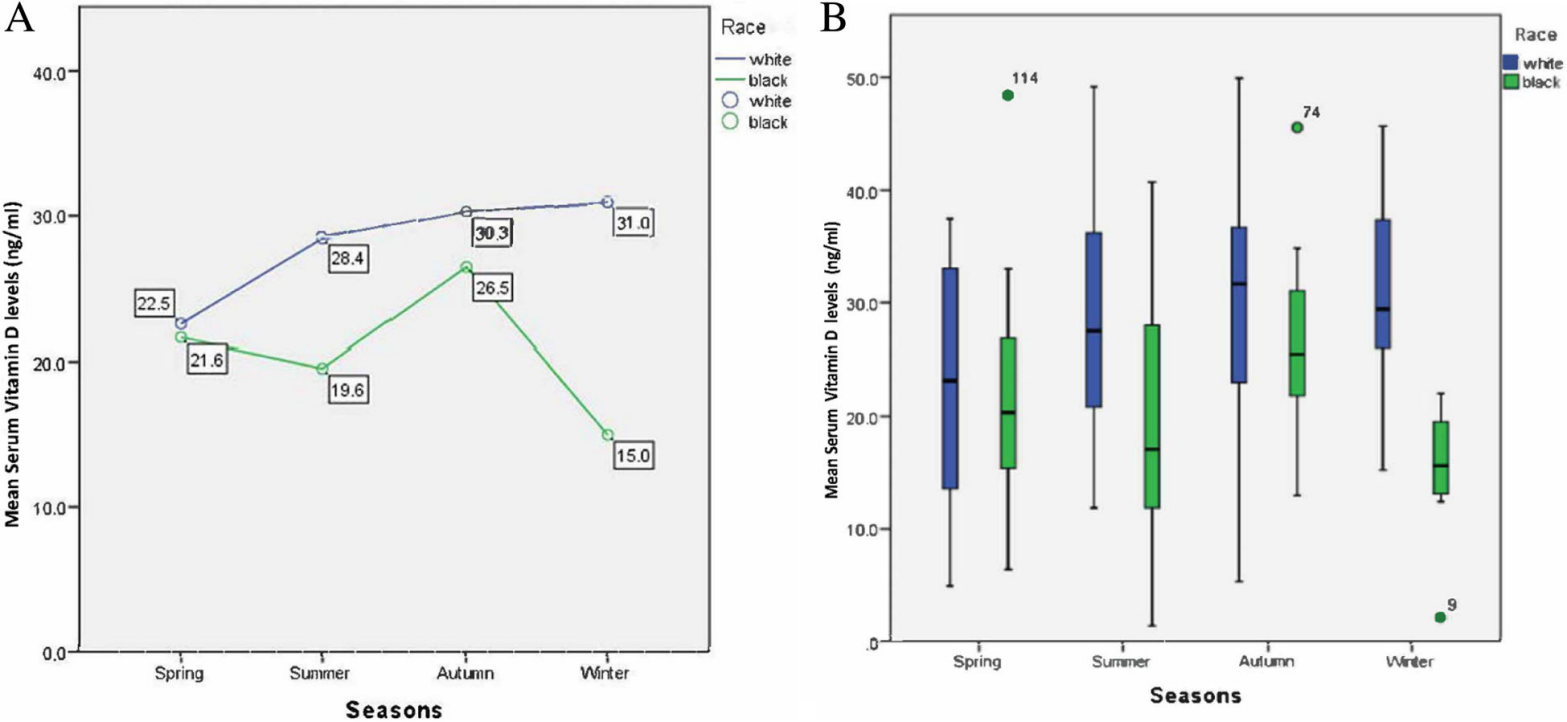
**a** and **b** The influence of race and seasonality on mean serum vitamin D_3_ levels in surveyed women

Predictor variables that influenced mean serum 25(OH) D_3_ independent of race include pregnancy, season, and cotinine status (Table 5). Overall, pregnant women had significantly higher mean serum 25(OH) D_3_ levels than non-pregnant women; however, when race is taken into consideration, we see that there was only a significant difference in mean serum 25(OH) D_3_ levels between White pregnant and White non-pregnant women. While the effect of season on mean serum 25(OH) D_3_ did not significantly interact with the effect of race on mean 25(OH) D_3_ (*p* = 0.067), a trend can still be observed (Fig. 5); Seasonality did not affect vitamin D status for White women (*p* = 0.130), but it did for Black women (*p* = .030). For Black women, the highest mean serum 25(OH) D_3_ levels were found in the autumn (26.5 ng/mL) and the lowest in the winter (15.0 ng/mL).

In the summer and winter White women had significantly higher mean serum 25(OH) D_3_ levels as compared to Black women.

Income, education, self-reported smoking levels, lead, mercury, cadmium exposure, and coastal residence had no statistically significant influence on the effect of race on vitamin D status, nor did they independently predict mean serum 25(OH) D_3_ levels.

## Discussion

Over one-third of all the women included in the study (38%) had severely deficient serum levels of vitamin D (< 20 ng/mL), 67% had levels below 30 ng/mL and the overall mean serum concentration was 24.1 ng/mL, suggesting that for a significant portion of low-income women living in southern Louisiana who were enrolled in the GROWH study, sun exposure, dietary intake, and supplementation do not provide adequate vitamin D support. These findings are similar to those of a National Health and Nutrition Examination Survey (NHANES), representative of 62 million US women between 2001 and 2006, showing that overall, 41% of US women aged 13–44 years had vitamin D deficiency (< 20 ng/mL), and 78% had levels < 30 ng/mL. The mean serum 25(OH)D concentration was 23.6 ng/mL [24].

The variables associated with mean 25(OH) D_3_ levels in this study were: race, season, pregnancy, tobacco exposure measured by salivary cotinine level and age. Pregnancy,  self-identifying as White, autumn season, young age and high exposure to tobacco smoke were associated with higher serum levels of vitamin D.

### Skin’s synthesis due to sun exposure

Cutaneous synthesis of vitamin D_3_ is mediated by skin pigmentation, latitude, season, time of day, and dermal exposure to the sun. The prevalence of vitamin D deficiency in this study was twice as high for Black women compared to White women, confirming prior studies that report much lower vitamin D levels among reproductive-aged women with more pigmented skin [10, 16, 24, 26, 43]. Deeply pigmented skin is very effective in absorbing UVB radiation and thus reducing the cutaneous synthesis of vitamin D_3_ [3] and is likely the major cause of the observed differences by race. It is possible that vitamin D status contributes to the reproductive health gap that exists between Black and White women, and improving vitamin D status in persons with deeply pigmented skin might help narrow this disparity [10, 16]. It is worth mentioning that research suggests that race-dependent differences in vitamin D binding protein (VDBP) could explain differences in total vitamin D levels with Blacks having less total vitamin D due to lower levels of VDBP [44]. However, Aloia et al. 2015 showed that only 4% of the observed race-associated differences in total 25(OH) D_3_ could be attributed to race dependent VDBP serum variations [45].

Seasonality affects vitamin D levels, with winter generally being the season with the lowest levels and summer the season with the highest [43, 46, 47]. During the winter months, sunlight is filtered at a more oblique angle, decreasing the ultraviolet radiation. Our study, however, showed that women sampled in autumn had the highest mean 25(OH) D_3_ levels. This observation coincides with previous research conducted among pregnant women in Louisiana, which demonstrated an atypical pattern of seasonal variation [28]. As in this presented data, Gangat et al. observed autumn to be the apex season for serum vitamin D levels in a similar study population. This atypical seasonal pattern of serum vitamin D levels may be linked to sun avoidance behaviors during the hot and humid summer months in southern Louisiana. Also, there is a high awareness of sun exposure-related skin cancer. Successful campaigns to control sun exposure through sunscreen use and sun avoidance behaviors are currently common in all racial groups and have, in combination with the adoption of lifestyles that have shifted to more time spent indoors, most likely led to increasing prevalence of vitamin D deficiency in the general population [10, 22]. Louisiana’s latitude and relatively high seasonal humidity as compared to other published study locations investigating seasonal variation in U.S. women such as Pennsylvania [46], Washington, North Carolina and Michigan [43] may explain an observed deviation from published season-mediated vitamin D fluctuations in similar research populations.

The more pronounced seasonal variation among Black women suggests sun avoidance behavior during the warm spring and summer seasons may be more common in that group. The large disparity between White and Black women in the winter suggests that the UVB radiation is inadequate in winter months for darker pigmented women to sustain serum vitamin D levels from sun exposure.

### Diet

Fish, especially oily-fish such as salmon, mackerel and bluefish, are considered a good source of vitamin D_3_. Counter to this, consumption of fish considered to be rich in vitamin D_3_, as well as fish and seafood types most frequently consumed by this population, had no influence on vitamin D_3_ levels in this study. There are multiple factors that could explain this. A meta-analysis by Lehman et al., 2015 investigating whether fish intake increases serum 25 (OH) vitamin D_3_ concentrations in healthy adults showed that the consumption of at least 2 fish meals, corresponding to approximately 300 g/week over a period of at least 4 weeks, was associated with a significant increase in 25(OH) vitamin D_3_ [48]. In the GROWH subpopulation of this study, fish consumption was analyzed in ounces per week (Table 1.) which shows that the majority of women (76%) consumed 3.5 oz (= 85 g) of fish or less per week.

The aforementioned meta-analysis also showed that the type of fish is an important factor, with fatty fish consumption improving vitamin D_3_ status. Fatty fish consumption was not popular in this population sample, which may explain why fish consumption had a non-significance influence over systemic vitamin D levels. The seafood most consumed by this study population was crustaceans such as shrimp which contains little to no vitamin D and therefore has little influence on overall serum vitamin D_3_ concentrations. Drewery et al., 2016 also found that while catfish and tilapia were regularly consumed among pregnant women in Louisiana, seafood sources with higher fatty acids content were consumed at a low frequency [49]. Chen et al., 2007 evaluated the effect of various cooking methods on the vitamin D_3_ content of food. While microwaving or baking has not been shown to decrease vitamin D_3_ content significantly, when frying salmon in vegetable oil, only about half of the vitamin D_3_ was recovered [50]. Fry cooking seafood in oil is a popular food preparation method in southeastern Louisiana.

Furthermore, Liu (2012) found that although mushrooms, eggs, and oily fish may potentially contain high concentrations of vitamin D_3_, these foods may provide an unreliable source of vitamin D_3_ or are not eaten in large enough amounts to significantly impact serum vitamin D status. Lui’s study, therefore, concluded that systemic vitamin D_3_ levels are primarily modulated by UV exposure and/or oral supplementation [51]. Although fish is one of the few foods that contain vitamin D_3_, there is still an ongoing discussion about whether fish intake contributes to a sufficient supply of vitamin D_3_ [48].

### Pregnancy

Higher 25(OH) vitamin D_3_ concentrations in pregnant women could be due to prenatal supplementation which is advised for pregnant women. While there is no data available for the GROWH participants on compliance to prenatal vitamin’s or on which type of prenatal vitamins were being used (and whether they contained vitamin D_3_), in similar populations, more than 90% of pregnant women reported using prenatal vitamins [34]. Although the focus of prenatal vitamins is predominantly on folic acid, iron, and calcium, most of them also contain around 400–600 IU vitamin D_3_ [25] and have shown to increase vitamin D status as pregnant women tend to have higher 25(OH) D_3_ levels than non-pregnant women [24, 52]. Ginde et al. 2010 showed that the mean 25(OH) D_3_ levels by increasing trimester were 46, 46, and 54 nmol/L, respectively, among women not taking vitamin D-containing supplements (*P* for trend = .27), compared to 61, 69, and 84 nmol/L among those taking supplements (*P* for trend < .001).

A randomized control trial by Hollis et al. 2011, examining the need, safety and effectiveness of vitamin D supplementation during pregnancy showed that, compared to the 400 IU group (control), a daily vitamin D dose of 2000 IU and 4000 IU was associated with improved serum vitamin D_3_ status throughout the pregnancy, one month prior, and at delivery in both mother and neonate [52].

Vitamin D binding protein (VDBP) could possibly be playing a role in higher serum 25 (OH) vitamin D_3_ levels in pregnant women. Because of the estrogen-dependent production of VDBP, pregnant women have been observed to have increased serum concentrations of vitamin D [53]. It is uncertain how much this innate biological response contributes to observed higher serum 25(OH) vitamin D_3_ levels in our study population.

### Other factors

Other influencing factors include tobacco exposure (measured by salivary cotinine), age and WIC participation. Interestingly, high cotinine (> 12.9 ng/mL) was associated with higher vitamin D_3_ levels for which we do not have a clear explanation. Possibly, the behavior of going outside to smoke and the strong influence of sun exposure on vitamin D_3_ synthesis might contribute to this observation. However, other research shows a negative correlation between smoking and vitamin D levels which could possibly be explained by the fact that smoking is usually accompanied by a less healthy lifestyle (less physical activity, alcohol consumption, and bad dietary habits) leading to reduced sun exposure and thus synthesis of vitamin D. A causative role of smoking in vitamin D deficiency could not be excluded but the mechanism that explains the effect of cigarette smoking in vitamin D metabolism remains unclear [40, 54, 55].

In other studies, younger age was associated with lower vitamin D_3_ levels in pregnancy, due to vitamin supplementation being more common among older women, suggesting that younger individuals may be less conscientious about health matters [26, 27]. Our study, however, showed higher serum 25(OH) D_3_ levels among the youngest age group (18–25 years). Older age might be related to more education and higher income, which have been associated with more employment indoors [26].

In White women, mean serum 25(OH) D_3_ levels abruptly decreased at age category 30–35, and a strong decrease was observed in Black women at age category > 35. Possibly, the high number of non-pregnant women in these age groups decreased the mean serum 25(OH) D_3_ concentrations. Non-pregnant women most likely have stopped taking prenatal vitamins as they are per definition labeled as “prenatal.”

While WIC participation was not significantly associated with vitamin D status in White women, Black women from WIC clinics had significantly higher serum vitamin D_3_ levels compared to Black women who were not recruited from WIC clinics. Although this study was not a controlled trial, this observation suggests that participation in the federally-funded WIC program improves nutrition-related health outcomes such as serum vitamin D_3_ deficiency in some populations of Black women. As an overall higher level of deficiency generally exists in Black women, small behavioral and dietary modifications may lead to relatively greater improvement in vitamin D levels. Although WIC participation has shown improvements in other outcomes such as infant mortality in Black/African American populations [56, 57], to our knowledge, this is the first study assessing the impact of WIC enrollment on serum 25(OH) D_3_.

A unique public health policy of universal vitamin D supplementations for all pregnant and lactating women and all children under 5 years of age, called the Heart of Birmingham Primary Care Trust, was implemented in Birmingham, UK in 2005 in response to an increasing resurgence of symptomatic cases of vitamin D deficiency in a high-risk predominantly ethnic minority population [58]. This universal program has reduced the cases of symptomatic vitamin D deficiency and has increased public awareness of vitamin D demonstrating that public health campaigns to tackle vitamin D deficiency through supplementation and nutritional education can be effective.

### Limitations

This study has several limitations that influence the generalizability of the results. This study was unable to collect data on daily sun exposure, sun avoidance behavior or outdoor physical activity. Although vitamin D (prenatal) intake was likely due to observed increased levels of serum vitamin D in pregnant women as compared to their non-pregnant counterparts, prenatal vitamin intake compliance was not quantified directly. Also, as a control for inter-racial differences in serum vitamin D binding protein, future studies might consider determining the role vitamin D binding protein has on serum vitamin D levels in similar populations. Although the 25(OH) vitamin D_3_ quantification methods utilized in this study are not certified by the Vitamin D Standardization Program (VDSP), immunoassay methods such as utilized in this study are employed by 97% of clinical laboratories. Also, given the cross-sectional and observational nature of the study, we were unable to draw hard conclusions about the observed effect such as vitamin D status improvement due to WIC enrollment.

Finally, the small sample size limited the statistical power and methods that could be applied to investigate interactions between multiple covariates. For example, the cotinine variable could not be incorporated into the multiple regression model due to an insufficient number of available samples.

## Conclusion

Vitamin D deficiency is common among low-income, reproductive-aged women living along the coast of southeastern Louisiana, which adds to a growing body of research showing inadequate serum vitamin D_3_ levels in reproductive-aged women worldwide. In this study, women at highest risk for vitamin D_3_ deficiency self-identified as Black and were non-WIC participants greater than 35 years of age. The winter season exacerbated vitamin D_3_ deficiency in this group of women. Interventions to improve vitamin D_3_ status are feasible and should be implemented to encourage dietary changes including healthy fish consumption, vitamin D_3_ supplementation, and safe sun exposure. Improving serum vitamin D_3_ status among this population may reduce adverse reproductive outcomes. Further studies are warranted to fully elucidate potential associations between serum vitamin D_3_ status and reproductive outcomes.

## Abbreviations

CDC: Centers for disease control
ELISA: Enzyme-linked immune sorbent Assay
EPA: US Environmental Protection Agency
GROWH: Gulf Resilience on Women’s Health
ICP-MS: Inductively coupled plasma-mass spectrometry
OSHA: Occupational Safety and Health Administration
PRAMS: Pregnancy Risk Assessment Monitoring
SGA: Small for Gestational Age
WIC: Women, Infants, and Children
